# Peripapillary gamma zone pit as dehiscence between Elschnig´s border tissue and Bruch´s membrane with herniation and defect of the retinal nerve fiber layer

**DOI:** 10.1186/s12886-016-0322-1

**Published:** 2016-08-12

**Authors:** Xinxin Hu, Yi Dai, Jost Jonas, Xinghuai Sun

**Affiliations:** 1 Department of Ophthalmology and Vision Science, Eye & ENT Hospital, Shanghai Medical College, Fudan University, Shanghai, China; 2 Key Laboratory of Myopia of State Health Ministry and Key Laboratory of Visual Impairment and Restoration of Shanghai, Shanghai, 20031 China; 3 Department of Ophthalmology, Medical Faculty Mannheim of the Ruprecht-Karls-University, Heidelberg, Germany

**Keywords:** Gamma zone pit, Peripapillary atrophy, Retinal nerve fiber layer defect, Suprachoroidal cavitation, Myopia, Case report

## Abstract

**Background:**

The parapapillary gamma zone has recently been defined as the parapapillary region free of Bruch’s membrane. Although it has been reported the presence of defects in peripapillary gamma zone, hitherto undescribed is the herniation of the retinal nerve fiver layer tissue into the peripapillary gamma zone defect with the resulting localized defects in the retinal nerve fiber layer.

**Case presentation:**

Ophthalmoscopy in a 36-year-old man revealed a localized defect of the retinal nerve fiber layer associated with a yellowish-gray lesion at the inferior temporal outer margin of a peripapillary gamma zone. Enhanced depth imaging of spectral-domain optical coherence tomography (OCT) showed a dehiscence at the connecting point between the central end of Bruch’s membrane and the peripheral end of the border tissue of Elschnig and Jacoby. Retinal nerve fiber layer tissue was herniated through this defect into a cavitation located in the suprachoroidal space and the space above the cerebrospinal fluid space. At a 2-year follow-up examination, the defect and retinal nerve fiber layer defect appeared unchanged.

**Conclusion:**

We present a peripapillary gamma zone pit originating as a dehiscence between Elschnig’s border tissue and Bruch’s membrane and associated with a herniation and defect of the retinal nerve fiber layer and with a suprachoroidal cavitation.

## Background

Previous studies have shown that the peripapillary region can be divided into a peripheral alpha zone, characterized by Bruch’s membrane covered with an irregularly structured retinal pigment epithelium; a beta zone showing Bruch’s membrane denuded of retinal pigment epithelium; and a gamma zone free of Bruch’s membrane [1]. Recent reports by Ohno-Matsui and our group showed the presence of defects in peripapillary gamma zone [2, 3]. Here we describe a patient who showed a peripapillary gamma zone defect with herniation and defect of the retinal nerve fiber layer.

## Case presentation

**Fig. 1:**
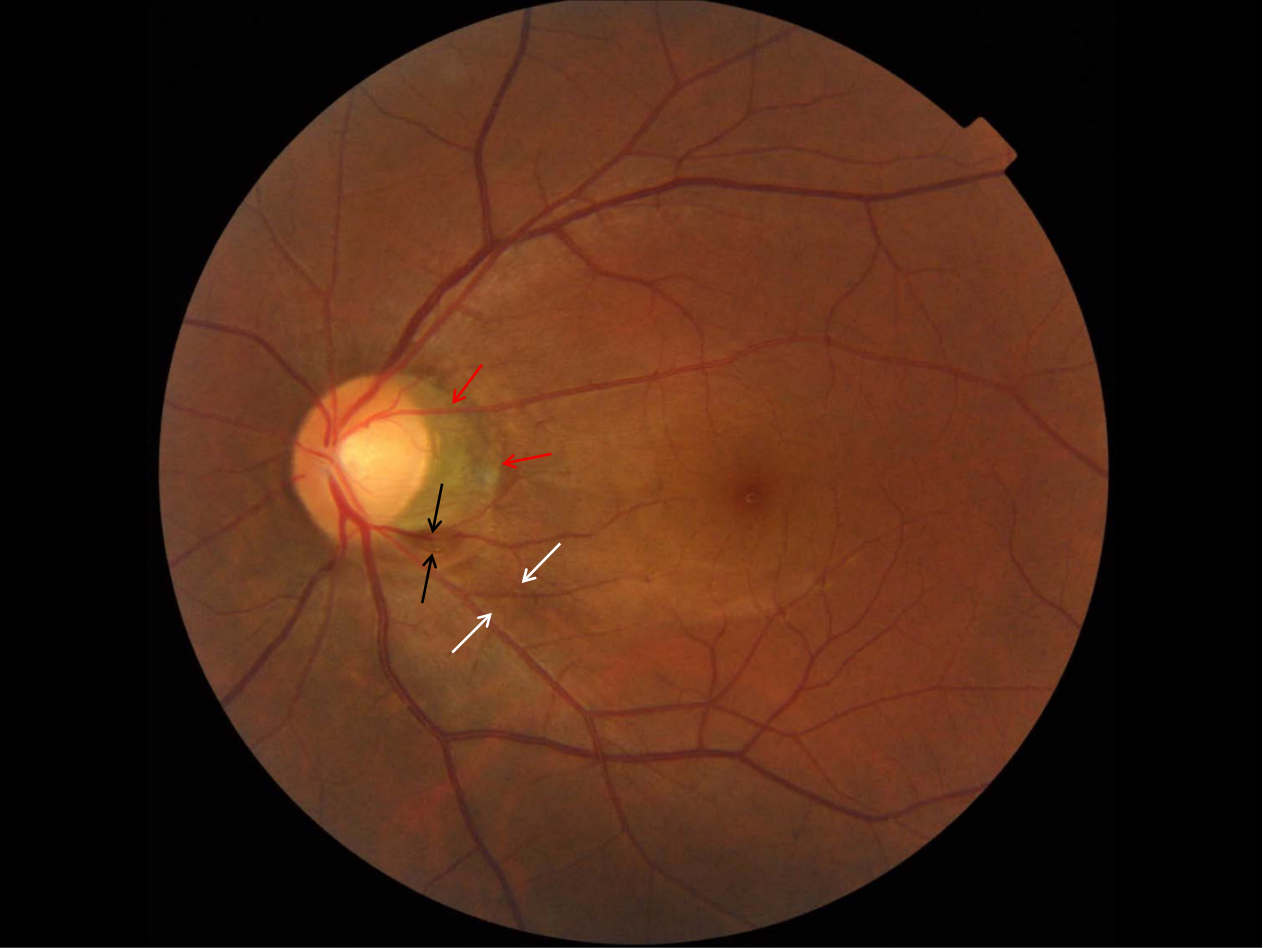
Color photograph of the left fundus. Color photograph of the left fundus, showing a localized retinal nerve fiber layer (*between both white arrows*), a yellowish-gray lesion (*between black arrows*) at the outer margin of the inferior temporal margin of a peripapillary gamma zone (*red arrows*)

**Fig. 2:**
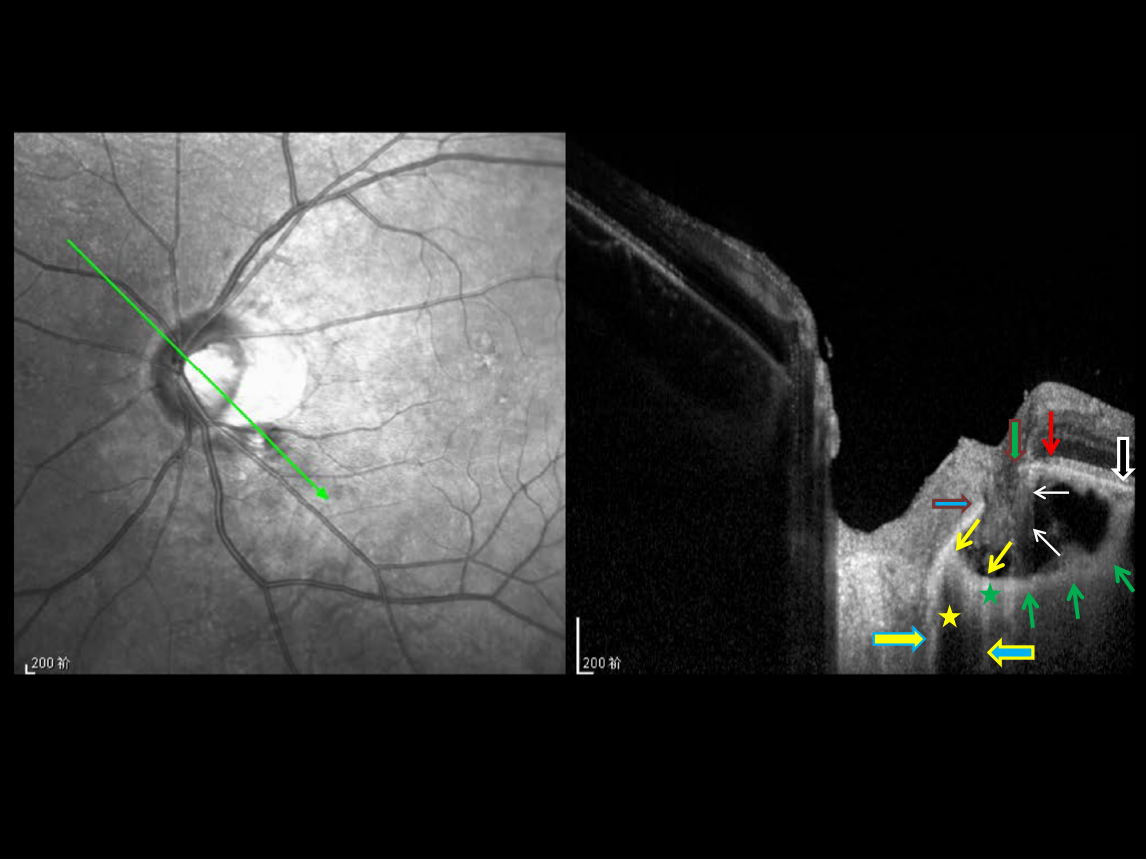
Oblique optical coherence tomographic image (enhanced depth imaging). Oblique optical coherence tomographic image (enhanced depth imaging), showing a defect between the central end of Bruch’s membrane (*vertical green arrow with red border*) and the peripheral end of the border tissue of Elschnig and Jacoby (*horizontal blue arrows with red border*), which normally separates the choroid from the intrapapillary region and connects between the central end of Bruch’s membrane and the pia mater of the optic nerve (*horizontal yellow arrow with blue borders*); retinal nerve fiber layer tissue is herniated (*white arrows*) into the suprachoroidal / supra-cerebrospinal fluid space cavitation; the cavitation is bordered by an elongated and thinned posterior sclera (green arrows) which connects with the peripapillary scleral flange (*between both yellow arrows*) at the merging point of optic nerve dura mater (*horizontal blue arrow with yellow borders*) with the sclera; the peripapillary scleral flange forms the roof of the cerebrospinal fluid space (*yellow star*) between the pia mater of the optic nerve (*horizontal yellow arrow with blue borders*) and the presumed dura mater (*horizontal blue arrow with yellow borders*); between vertical green arrow with red borders and the vertical red arrow: peripapillary alpha zone with presence of Bruch’s membrane and presence of irregularly structured retinal pigment epithelium; there is no peripapillary beta zone since Bruch’s membrane (*vertical black arrow with white borders*) is covered with retinal pigment epithelium (normal or irregularly structured) all along its course

**Fig. 3:**
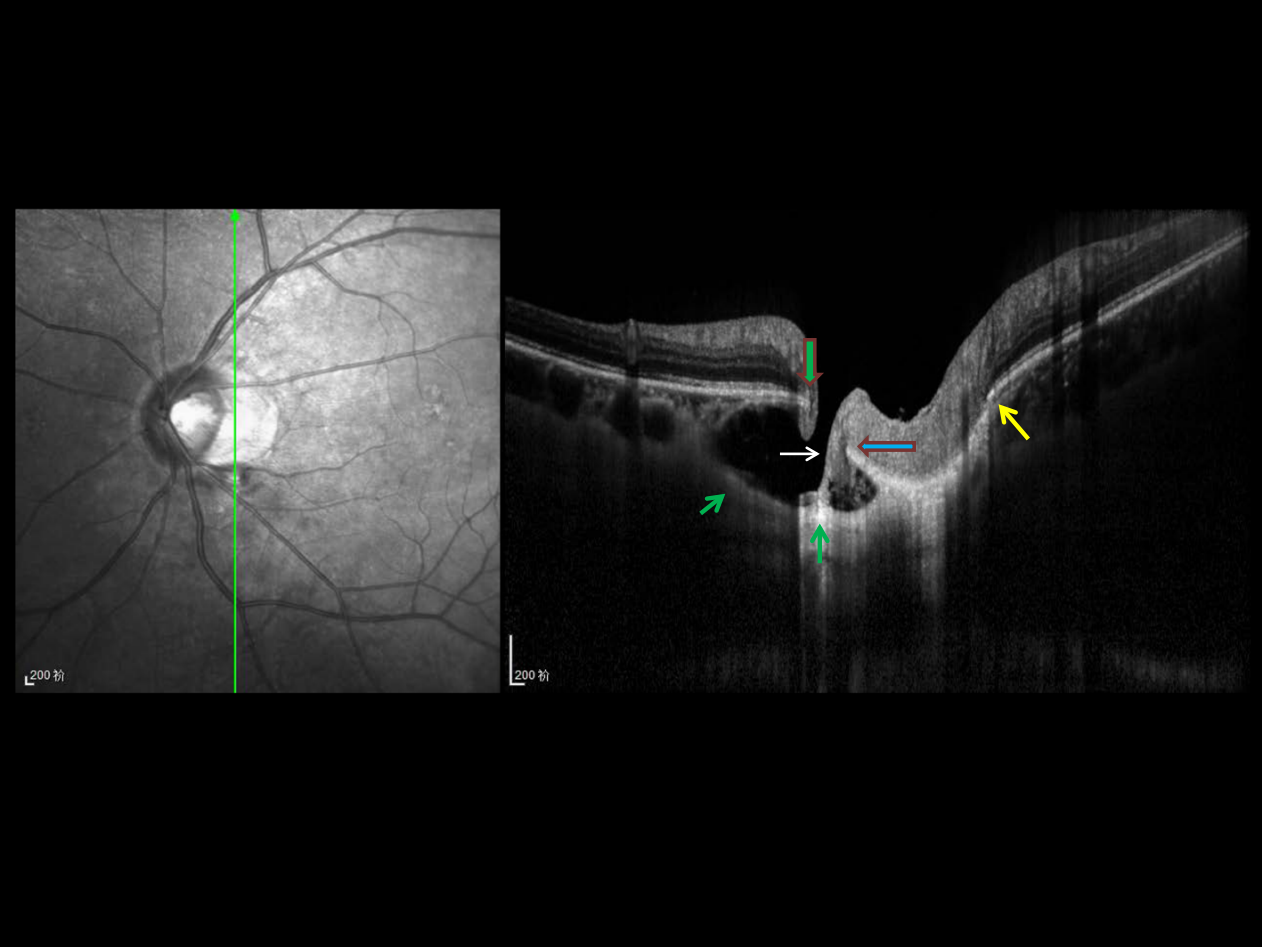
Vertical optical coherence tomographic image (enhanced depth imaging). Vertical optical coherence tomographic image (enhanced depth imaging), showing a defect between the central end of Bruch’s membrane (*vertical green arrow with red border*) and the peripheral end of the border tissue of Elschnig and Jacoby (*horizontal blue arrows with red border*), allowing the herniation of retinal nerve fiber layer tissue (*white arrows*) into the suprachoroidal / supra-cerebrospinal fluid space cavitation; the cavitation is bordered by an elongated and thinned posterior sclera (*green arrows*); yellow arrow: contralateral end of Bruch’s membrane and marking the beginning of peripapillary gamma zone (Bruch’s membrane free, peripapillary zone)

**Fig. 4:**
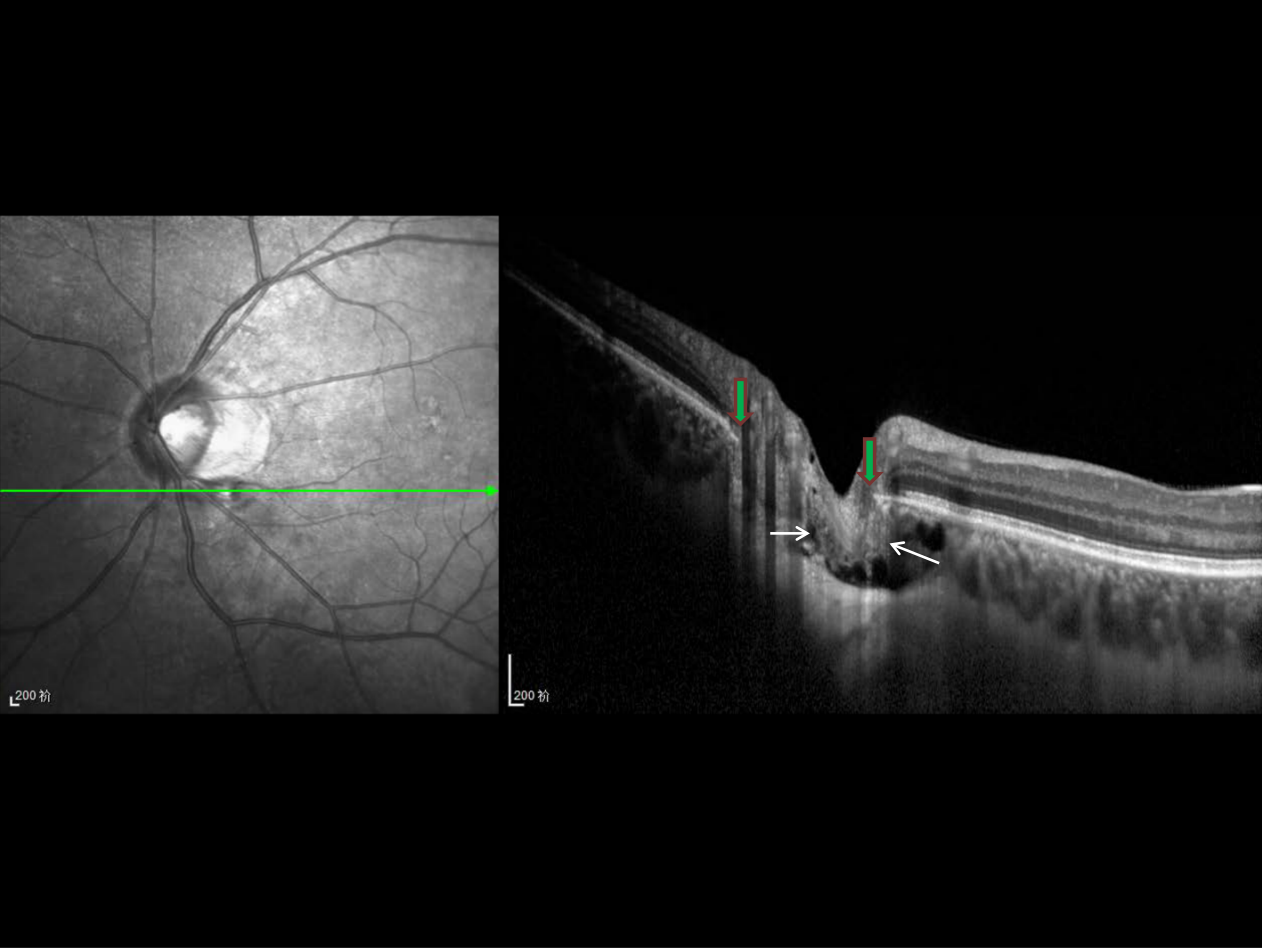
Horizontal optical coherence tomographic image (enhanced depth imaging). Horizontal optical coherence tomographic image (enhanced depth imaging), showing the herniation of retinal nerve fiber layer tissue (*white arrows*) between the two ends of Bruch’s membrane (*vertical green arrows with red border*) into the suprachoroidal / supra-cerebrospinal fluid space cavitation

A 36-year-old, highly myopic man (refractive error, OD:−6.00 = −1.00/180°; OS: −5.50 = −0.75/180°) with best corrected visual acuity of 20/20 OU, axial length of 27.5 mm (OD) and 27.11 mm (OS), and normal intraocular pressure, showed upon ophthalmoscopy in his left eye a localized defect of the retinal nerve fiber layer in the inferior temporal region (Fig. 1). The localized retinal nerve fiber layer was spatially associated with a yellowish-gray lesion at the inferior temporal outer margin of a peripapillary gamma zone (Fig. 1). Enhanced depth imaging of spectral-domain optical coherence tomography (OCT) revealed a dehiscence at the connecting point between the central end of Bruch’s membrane and the peripheral end of the border tissue of Elschnig and Jacoby. The latter normally separates the choroid from the intrapapillary region and extends from the central end of Bruch’s membrane to the pia mater of the optic nerve (Fig. 2). Retinal nerve fiber layer tissue was herniated through this defect into a cavitation located in the suprachoroidal space and the space above the cerebrospinal fluid space (Figs. 2, 3 and 4). The cavitation was bordered by an elongated and thinned posterior sclera connecting with the peripapillary scleral flange at the merging point of the optic nerve dura mater with the sclera (Fig. 2 and 3). At a 2-year follow-up examination, the defect and retinal nerve fiber layer defect appeared unchanged.

## Conclusions

Hitherto undescribed is the herniation of the retinal nerve fiver layer tissue into the peripapillary gamma zone defect with the resulting localized defects in the retinal nerve fiber layer. It has remained unclear whether a partial blockade of the axoplasmic flow in the nerve fibers by the herniation caused the drop-out of the fibers. Also, in contrast to the patients with peripapillary pits described by Ohno-Matsui et al., who were more myopic (−9.5D to −22.0D) and had longer axial lengths (29.5 mm to 32.8 mm), our patient was not that highly myopic [2]. The finding in our patient may suggest a peripapillary gamma zone pit can develop by a dehiscence between the border tissue of Elschnig and Bruch’s membrane at their connecting point. In our patient, peripapillary gamma zone defect may not have been caused by a schisis in the sclera or in spatial correlation with an opening of a short posterior ciliary artery as described previously [1, 3].

In conclusion, a peripapillary gamma zone pit was presented originating as a dehiscence between Elschnig’s border tissue and Bruch’s membrane and associated with a herniation and defect of the retinal nerve fiber layer and with a suprachoroidal cavitation.

## Abbreviations

OCT, optical coherence tomography

## References

[1] Jonas JB, Jonas SB, Jonas RA, Holbach L, Dai Y, Sun X, Panda-Jonas S (2012). Parapapillary atrophy: histological gamma zone and delta zone. PLoS ONE.

[2] Ohno-Matsui K, Akiba M, Moriyama M, Shimada N, Ishibashi T, Tokoro T, Spaide RF (2012). Acquired optic nerve and peripapillary pits in pathologic myopia. Ophthalmology.

[3] Dai Y, Jonas JB, Ling Z, Sun X (2013). Parapapillary gamma zone hole. J Glaucoma.

